# Staging in Gerodontology: A Framework

**DOI:** 10.1111/ger.70058

**Published:** 2026-05-29

**Authors:** Falk Schwendicke, Gerry McKenna, Murali Srinivasan, Azize Bell, Najla Chebib, Cristiane Da Mata, Helena Dujic, Jennifer Gallagher, Barbara Janssens, Danielle M. Layton, Manabu Kanazawa, Anastassia Kossioni, Sabrina Maniewicz, Koichiro Matsuo, Pedro Molinero Mourelle, Annika Neri, Takahiro Ono, Sungkrit Pojmonpiti, Foteini Spyraki, Kittipit Srisanoi, Saif S. Syed, Sayaka Tada, Georgios Tsakos, Terry R. Walton, W. Murray Thomson, Jean‐Philippe Haesler, Sophie Dartevelle, Frauke Müller

**Affiliations:** ^1^ Department of Conservative Dentistry, Periodontology and Digital Dentistry, LMU University Hospital LMU Munich Munich Germany; ^2^ Centre for Public Health Queen's University Belfast Belfast UK; ^3^ Clinic of General‐, Special Care‐ and Geriatric Dentistry, Center for Dental Medicine University of Zurich Zurich Switzerland; ^4^ Department of Physiology, Faculty of Dentistry, Center of Excellence in Precision Medicine and Digital Health Chulalongkorn University Bangkok Thailand; ^5^ Universitätsmedizin der Johannes Gutenberg‐Universität Mainz Poliklinik für Zahnärztliche Prothetik und Werkstoffkunde Mainz Germany; ^6^ Division of Gerodontology and Removable Prosthodontics, University Clinics of Dental Medicine University of Geneva Geneva Switzerland; ^7^ Restorative Dentistry Cork University Dental School and Hospital, UCC Cork Ireland; ^8^ Faculty of Dentistry, Oral & Craniofacial Sciences King's College London London UK; ^9^ ELOHA (Equal Lifelong Oral Health for All) Research Group, Department of Oral Health Sciences, Faculty of Medicine and Health Sciences Ghent University Ghent Belgium; ^10^ Department of Oral Health Ghent University Hospital Ghent Belgium; ^11^ School of Dentistry, Faculty of Health and Behavioural Sciences University of Queensland St Lucia Queensland Australia; ^12^ Specialist Private Practice St Lucia Queensland Australia; ^13^ Gerodontology and Oral Rehabilitation, Graduate School of Medical and Dental Sciences Institute of Science Tokyo Tokyo Japan; ^14^ Discipline of Gerodontology, Department of Prosthodontics, Dental School National and Kapodistrian University of Athens Psachna Greece; ^15^ Department of Oral Health Sciences for Community Welfare, Graduate School of Medical and Dental Sciences Institute of Science Tokyo Tokyo Japan; ^16^ Department of Reconstructive Dentistry and Gerodontology, School of Dental Medicine University of Bern Bern Switzerland; ^17^ Department of Conservative Dentistry and Orofacial Prosthodontics, Faculty of Dentistry Complutense University of Madrid Madrid Spain; ^18^ Complutense University of Madrid, Ramon y Cajal Research Institute (IRYCIS) Madrid Spain; ^19^ Department of Geriatric Dentistry Osaka Dental University Osaka Japan; ^20^ Department of Neurology Ghent University Hospital Ghent Belgium; ^21^ Dental Department, Chulabhorn Hospital Chulabhorn Royal Academy Bangkok Thailand; ^22^ Faculty of Dentistry Khon Kaen University Khon Kaen Thailand; ^23^ Department of Epidemiology and Public Health University College London London UK; ^24^ Institute of Dentistry Queen Mary University of London London UK; ^25^ Faculty of Dentistry National University of Singapore Singapore; ^26^ Department of Epidemiology and Public Health University College London (UCL) London UK; ^27^ School of Dentistry, Faculty of Medical Sciences University of Sydney Sydney New South Wales Australia; ^28^ Specialist Private Practice Sydney New South Wales Australia; ^29^ Faculty of Dentistry University of Otago Dunedin New Zealand; ^30^ Swiss Dental Association SSO Bern Switzerland; ^31^ The European Regional Organization of the FDI World Dental Federation ERO‐FDI Gümligen Switzerland

**Keywords:** case complexity, frailty, geriatric dentistry, hyposalivation, nutrition, older adults, oral health care, socio‐economic status, treatment planning, xerostomia

## Abstract

**Introduction:**

Comprehensively, systematically and reproducibly assessing and describing the (oral) health status and care needs of older adults is challenging. We aimed to provide a framework for evaluating and reporting older adults' oral health, capturing general and oral function and disease, as well as oral, systemic and socio‐economic/social risk factors determining oral care complexity and capability, care needs and levels, and clinical management, including prevention, active and supportive therapy, as well as informing education, research, surveillance and health services. The present paper describes the first aspect, staging of oral health in this population.

**Methods:**

Eight reviews were conducted to identify risk factors for deterioration of oral health in older adults; existing systematic reviews were further considered during the framework development. A 2.5‐day workshop with 31 international researchers and stakeholders was held and a structured consensus process implemented to come to an agreed, internationally applicable framework. A 75% level of agreement was achieved after two rounds of voting.

**Results:**

A case definition based on staging and grading, reflecting the current general and oral health and function, care complexity, and the risk of deterioration of oral health due to local, systemic, and socio‐economic and social factors, was developed. Staging includes person‐level (based on clinical frailty), function‐level (based on clinical and subjective evaluations reflecting oral function, such as tooth loss and oral impacts), and disease‐level (including caries, periodontal disease, and oral mucosa assessments) domains.

**Conclusion:**

Oral health staging in older adults can allow clinicians, carers, and policymakers to systematically and comprehensively define and classify patient cases in order to derive appropriate and adequate care. Staging should be used in combination with grading, and both may allow capturing the overall health status and care needs of older patients for health surveillance, research, and education purposes. Next steps will include validation, dissemination, and implementation of the framework.

## 1 Introduction

Several problems in defining the oral health status and care needs of older adults have been identified. First, gerodontology addresses the oral health problems of older people with various levels of care dependency, including the frail and care dependent ones. This implies that often gerodontology is about pragmatic‐only ‘herodontics’, covered by enthusiastic professionals. Notably, there has been a call for the profession to focus on preventive approaches and effective and targeted interventions for middle age and care independent older adults minimising rapid oral health deterioration at the onset of dependency, particularly considering the changing demographics in most countries toward an ageing society in parallel to the epidemiological transition in oral health of older adults that is characterised by care needs emanating from past (mainly restorative and complex prosthetic) care. Second, remuneration systems do not account for the multi‐level character of oral care provision for older adults. Current funding models either do not adequately support any dedicated interventions for this group or only provide a one‐size‐fits‐all package of services to a group understood as always requiring ‘intensive’ care, ignoring the considerable heterogeneity in both oral and general health, and also biological ageing, risk factors, context, and expectations in terms of oral health and function. Moreover, many dental services operate in separation from other geriatric care teams, with limited integration of dental into wider (interdisciplinary) care models. Third, this heterogeneity and complexity lead to a wide range of challenges related to the available options for prevention, diagnosis and therapy in this population. These are usually addressed in isolation within dental education and treatment paradigms, without adequate consideration of their interdependency. Currently, it is not easy to include this interdependent set of factors when determining the needs and options for care.

Overall, there is currently only a limited focus on comprehensively, systematically, and reproducibly assessing and describing the oral healthcare status, care needs, and risk factors for oral health deterioration in older adults. These limitations in assessment and description have implications at various levels. While a range of individual systems and initiatives have attempted to provide a framework for targeting such assessment and description, none of these have so far gained popularity and reached international acceptance. Further complexity arises from the involvement of various groups of health professionals, such as physicians, nurses, dental hygienists, dentists, and also carers. The main focus of existing screening tools is identifying referral and care need, whereas tools to define care complexity, like the ones for people with dementia, are limited. The current work aimed to develop and agree on a framework to comprehensively assess and describe older adults' oral health, capturing general and oral function and disease (staging), as well as oral, systemic, and socio‐economic/social risk factors determining oral care complexity and capability, care needs and levels, and clinical management (grading). The present paper focuses on staging and should be used complementarily to the one on grading. The developed framework is expected to benefit clinical care, but also education, research, and public health. Notably, the framework was not developed based on any theoretical model, but on systematically compiled data and expert consensus (see below). Moreover, it has not been validated and does not always recommend specific instruments to be employed for determining individuals' stage and grade. The assignment, or development and validation of such instruments, and, generally, the implementation of the framework are planned for the future.

## 2 Methods

### 2.1 Process

A steering committee (F.S., M.S., G.M., and F.M.) of four individuals oversaw the project and initiated a scoping of existing case definitions and approaches in classifying older adults as part of their oral health assessment. The committee conducted a 1‐day workshop in December 2024 in Munich, Germany, and, during that workshop, agreed that ‘staging and grading’ would be a valuable option for case definition and classification. It then outlined that a consensus process, supported by systematically compiled evidence, would best be suited to develop and agree on the framework. The evidence sought should be as broad as possible and, ideally, carry most aspects of the framework, while the consensus process was to be globally inclusive, involving a broad spectrum of participants, as well as stakeholders from various Associations (European College of Gerodontology ECG, International Association of Dental Research IADR, World Dental Federation FDI, International Association of Dental Hygienists IADH, European Geriatric Medicine Society EuGMS, Swiss Dental Association SSO).

### 2.2 Aspects

While developing and discussing the framework, key aspects relevant to staging were identified:

1. An individual's general health status, which impacts on oral health, care complexity and self‐care.
2. Oral function, including the number of teeth present and subjective measures of function (eating, smiling, speaking, and comfort).
3. The presence, severity, and extent of common or debilitating oral conditions, including dental, periodontal, and mucosal conditions.

These were to be considered in the development and definition of the framework. A range of reviews were commissioned to support developing the framework (Table 1), all informing the development of both staging and grading criteria.

**TABLE 1:** Reviews conducted to support the development of the framework.

Authors	Review title
Falk Schwendicke, Katrin Heck, Julia Schwärzler, Cristiane da Mata, Sayaka Tada, Helena Dujic	Coronal and Root Caries in Older Adults: Associated Factors, Quality of Life Impact, and Treatment—A Scoping Review [1]
Gerry McKenna, Lewis Winning, Y de Waal, Kristina Bertl, Rawan Kahatab, Peter Harrison, Ioannis Polyzois	The Impact of Periodontitis on Oral Health Outcomes in Older Adults: A Systematic Review [2]
Murali Srinivasan, Murray Thomson, Porawit Kamnoedboon, Foteini Spyraki	A Systematic Review and Meta‐Analysis of the Global Prevalence of Dry Mouth in Older Adults [3]
Pedro Molinero, Takahiro Ono, Manabu Kanazawa, Najla Chebib, Azize Bell, Frauke Müller	Functional Outcomes of Prosthetic Rehabilitation in Older Adults With Tooth Loss: A Systematic Review and Meta‐Analysis [4]
Gerry McKenna, Finbarr Allen, C. Moore, A. Willis, N. Walls	Adverse Oral Health in Older Adults With Head and Neck Cancer (And Oral Potentially Malignant Disorders): A Systematic Review [5]
Murali Srinivasan, Claudio Leles, Frauke Müller, Gerry McKenna, Sabrina Maniewicz, Kittipit Srisanoi, Ines Wnuk	Associations Between Multimorbidity, Polypharmacy and Oral Conditions in Older Adults: A Systematic Review and Meta‐Analysis [6]
K. Srisanoi, I. Wnuk, S. Maniewicz, G. McKenna, F. Müller, C. Leles, P. Papi, J. Woodside, M. Srinivasan	The Impact of Nutritional Status on Oral Health Outcomes and Management in Older Adults: A Systematic Review and Meta‐Analysis [7]
Saif S. Syed, Sungkrit Pojmonpiti, Barbara Janssens, Murali Srinivsan, Frauke Müller, Georgios Tsakos	Association of Social Support With Oral Health in Older Adults: A Systematic Review and Meta‐Analysis [8]
Sungkrit Pojmonpiti, Saif S. Syed, Murali Srinivasan, Georgios Tsakos, Frauke Müller, Barbara Janssens	Association of Socioeconomic Factors With Oral Health in Older Adults: A Systematic Review and Meta‐Analysis [9]

### 2.3 Consensus Conference and Voting

Snowball sampling was conducted to contact individuals and invite them to participate in the consensus conference. Overall, 31 individuals from a total of 13 countries from Australia, Belgium, Brazil, France, Germany, Greece, Ireland, Japan, New Zealand, Switzerland, Singapore, Thailand, and the United Kingdom participated. The consensus group included academics, clinicians, researchers, methodologists, journal editors, regulatory professionals, and policymakers. Most stakeholders were familiar to the organisers. Before the consensus process and Delphi, participants were given written information about the study and were provided with a range of documents developed beforehand, including the commissioned reviews (in various stages). Participants ranged in age from 26 to 70, and 15 of them were women.

The consensus conference was held in Geneva on 26th–28th August 2025. A 60 min presentation on the reviews and the project itself, its aims, the findings of the reviews and the draft recommendations formed the basis for the consensus conference and the onsite voting process started off the consensus conference. Participants then discussed the process and its aims, as well as the findings of the reviews in a first plenum that lasted for approximately 3 h. In three subsequent subgroup sessions, the grading processes were discussed (each with 9–11 participants), on an oral, systemic, and social level. These discussions were followed by two further plenary sessions that allowed an in‐depth debate on the developed documents and the framework itself. Finally, participants anonymously voted on each statement (Table 2). The rules for the voting process were agreed on at the first plenary; that is, before the subsequent workshop proceedings.

**FIGURE 1:**
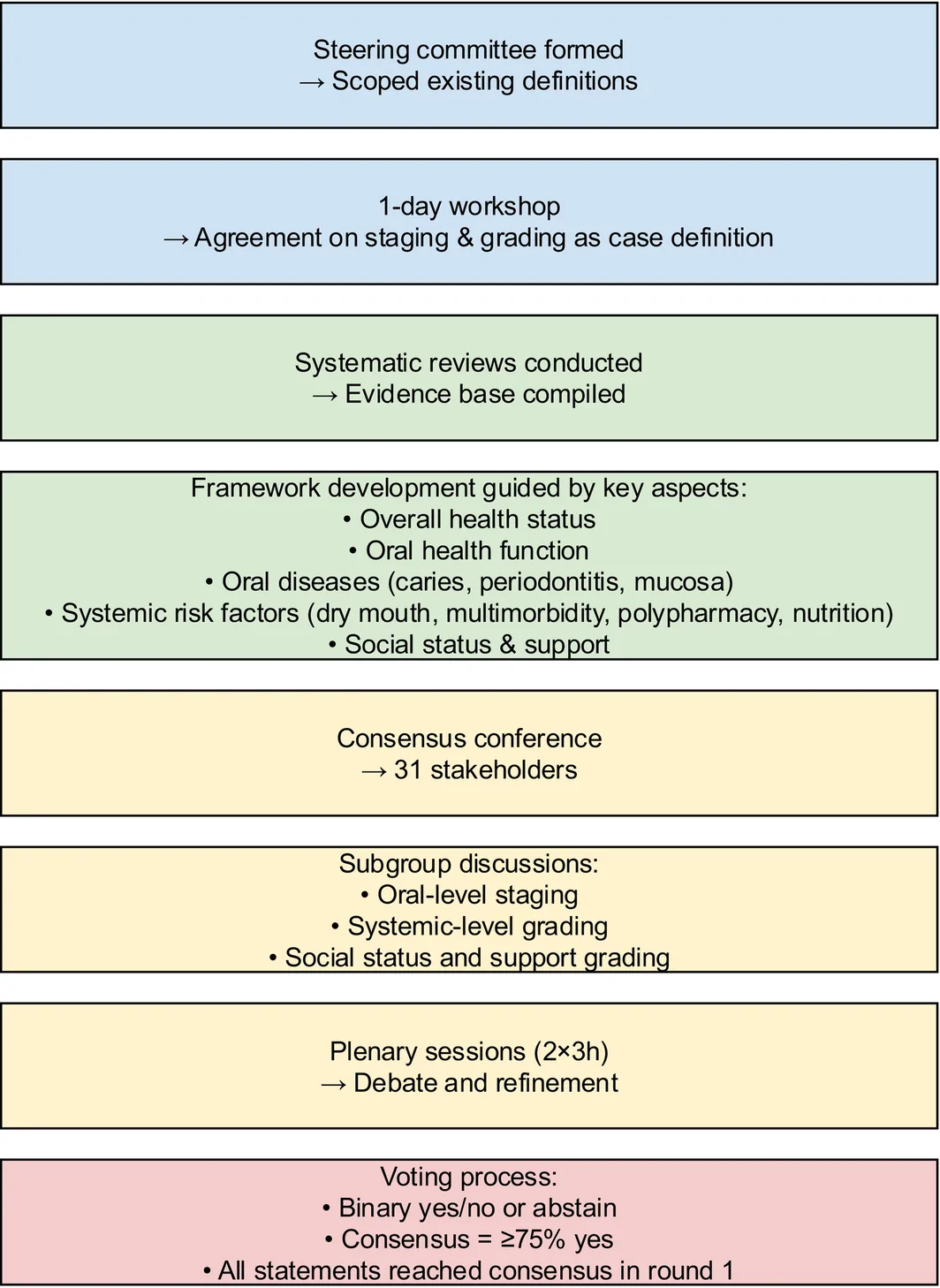
Process of developing the staging and grading framework for oral health in older adults. [Colour figure can be viewed at wileyonlinelibrary.com]

The voting (using Mentimeter; Stockholm, Sweden, https://www.menti.com) mandated the participants to vote ‘yes’ or ‘no’ (binary) or to abstain. Participants could also comment on each item. It was predefined that a statement achieved consensus when 75% or more of all participants agreed on it (abstentions were counted as no agreement). Note that not all people were present when each item was voted on, resulting in 27–30 votes being provided. Two rounds of voting were planned to allow discussion on items which may have not received agreement in the first voting round, followed by a second round of voting. All items received agreement in the first round, though. Reporting adheres to the Guidance on conducting and reporting Delphi studies (CREDES) [10]. The flow of methods is summarised in Figure 1.

## 3 Results

The group agreed that staging and grading were best suited to comprehensively and systematically reflect on a person's frailty, function of the masticatory system, the presence, severity, extent, complexity and progression of certain oral diseases, and the presence of systemic health conditions, along with social status and social support determining the ability to receive professional oral care or undertake self‐care. Staging and grading were assumed to capture an individual's oral health status and function, risk factors for rapid oral health deterioration and complexity of management needs, whether applicable for education and research, or for surveillance purposes, and to complement health services and policy development. Specifically, it was assumed that such an approach allowed assessors to: (1) systematically and comprehensively define patient case complexity; (2) derive primary preventive efforts and determine active and supportive care; (3) link up with the International Classification of Functioning, Disability and Health; (4) define value bases for management and remuneration; and (5) capture the general health status and care needs of older adults.

**FIGURE 2:**
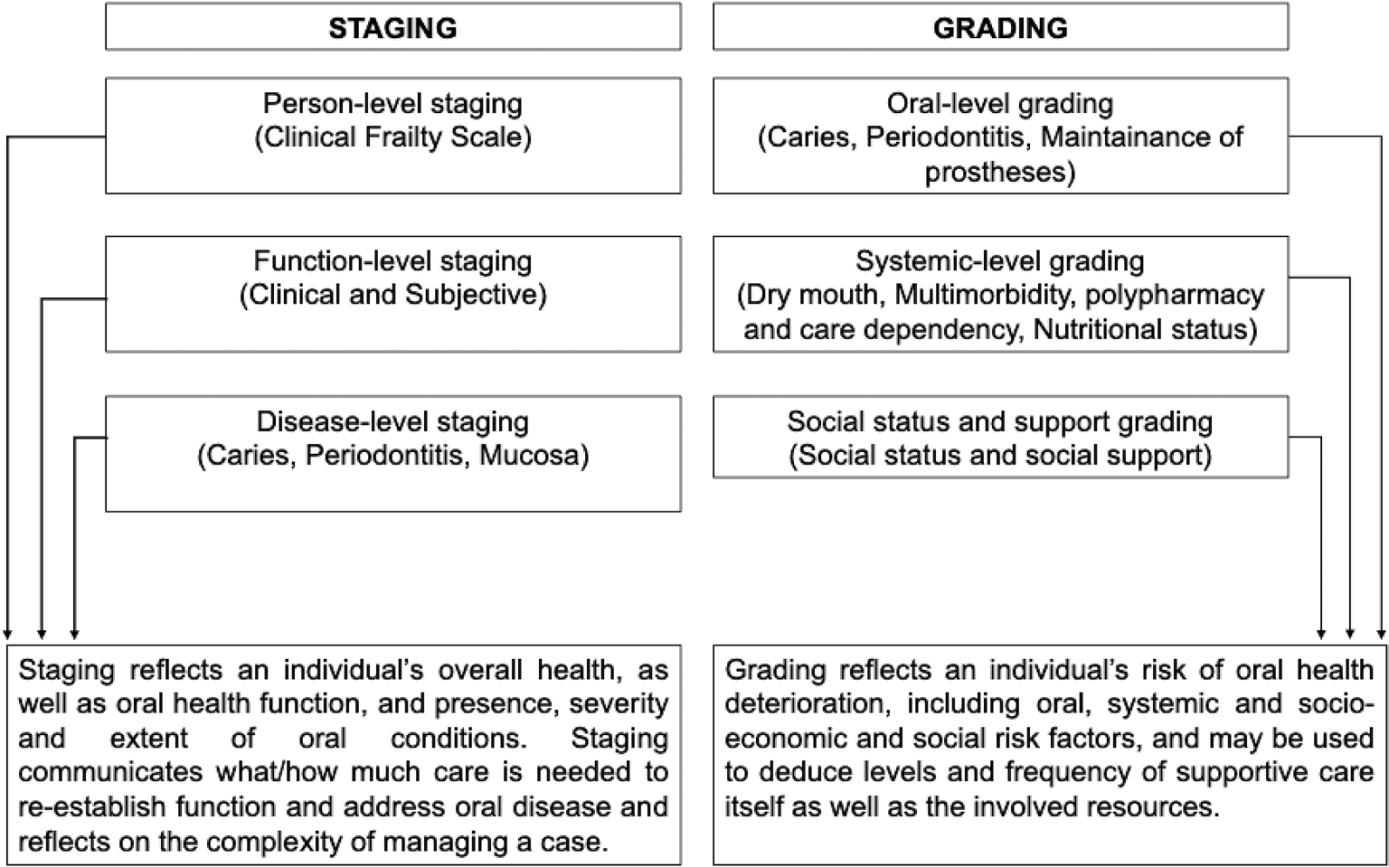
Flowchart guiding staging and grading.

The framework's application is not limited to specific age groups, since the group of older adults has a varying definition, and relevant aspects (including frailty) may also apply to younger groups. Figure 2 and Table 2 summarise the resulting staging and grading framework. Staging reflects the general complexity of managing a case given a patient's level of function and general health. Grading assesses risk factors for future oral health deterioration, including oral, systemic and social factors, and it may be used to determine the levels and frequency of supportive care as well as the resources required. The agreement (yes votes per all votes or abstentions) for each item is indicated. We here focus on staging only; grading is described in parallel elsewhere.

**TABLE 2:** Aspects to consider during staging and grading.

Domain	Stage (I–III)	Grade (A–C)
Person‐level	Frailty: CFS 1–3 = Stage I; CFS 4–6 = Stage II; CFS 7–9 = Stage III	—
Function‐level (clinical)	Stage I = shortened dental arch; Stage II = less than shortened arch but bilateral occlusal support; Stage III = no bilateral occlusal support	—
Function‐level (subjective)	Stage I = no oral impact; Stage II = one oral impact; Stage III = > 1 oral impact/no answer	—
Caries	Stage I = no active lesions; Stage II = active lesions w/o pulpal involvement; Stage III = active lesions w/pulpal involvement/infection	Grade A = no active caries/no risk factors; Grade B = no active caries but risk factors; Grade C = active caries or high risk
Periodontitis	Stage I = PPD ≤ 4 mm, no mobility II/III; Stage II = PPD > 4 mm, no mobility II/III; Stage III = PPD > 4 mm + mobility II/III	Grade A = minimal progression (%BL/age < 0.25, no bone loss in 5 years); Grade B = moderate progression (%BL/age 0.25–1); Grade C = rapid progression (%BL/age > 1, ≥ 2 mm loss in 5 years)
Mucosa	Stage I = healthy; Stage II = non‐severe lesion (e.g., candidiasis, pressure ulcer); Stage III = severe/potentially malignant lesion, incl. MRONJ etc.	—
Prostheses	—	Grade A = no prosthesis; Grade B = prosthesis well‐designed, manageable; Grade C = prosthesis defective, poorly fitting, or not manageable for patient
Dry mouth	—	Grade A = no/mild dry mouth (normal flow, negative mirror test); Grade B = moderate xerostomia or SGH; Grade C = severe xerostomia or SGH (mirror sticks, very low flow)
Multimorbidity/polypharmacy/dependency	—	Grade A = < 5 meds, no xerogenic drugs, CFS 1–3, no major geriatric conditions; Grade B = 5–9 meds or xerogenic drugs or CFS 4–6; Grade C = ≥ 10 meds or CFS 7–9 or major geriatric conditions
Nutrition	—	Grade A = normal MNA ≥ 24/MNA‐SF ≥ 12, no oral limitations; Grade B = at risk (MNA 17–23.5/MNA‐SF 8–11) or oral limitations; Grade C = malnourished (MNA < 17/MNA‐SF ≤ 7, calf circ. < 31 cm)
Social status	—	Grade A = high status (MacArthur 8–10, high income, tertiary education); Grade B = medium (MacArthur 3–7, medium income, secondary education); Grade C = low (MacArthur 0–2, low income, primary or lower education)
Social support	—	Grade A = not living alone; Grade B = living alone with support; Grade C = living alone without support

Abbreviations: BL, bone loss; CFS, Clinical Frailty Scale; MNA, Mini Nutritional Assessment; MRONJ, medication‐related osteonecrosis of the jaw; PPD, probing pocket depth; SGH, salivary gland hypofunction.

### 3.1 Staging

#### 3.1.1 Person‐Level Staging (Agreement: 30/30)

*Person‐level staging* reflects an individual's overall clinical frailty status and provides the basis for estimating oral care management complexity. Frailty is a central determinant of health trajectories in older adults, influencing not only the onset and progression of oral diseases but also the ability to engage in self‐care, access dental care, and tolerate active or supportive therapies. By categorising older adults according to their score on the *Clinical Frailty Scale* (*CFS*), the framework ensures that patient case definitions are sensitive to differences in resilience, independence, and vulnerability. The CFS is validated, widely used, and can be applied in both clinical and research settings, making it both a valid and practical tool for routine implementation [11].

**Table:** 

Stage	Criteria
Stage I	Clinical Frailty Scale 1, 2 or 3
Stage II	Clinical Frailty Scale 4, 5 or 6
Stage III	Clinical Frailty Scale 7, 8 or 9

Guideline for application

- *Scope of use*: Person‐level staging should be applied at the beginning of the assessment process, because it sets the overall context for evaluating oral health and care complexity.
- *Procedure*: Determine the individual's frailty stage using the Clinical Frailty Scale and assign the corresponding stage (I–III). This provides a standardised entry point for case definition and further staging/grading within the framework.

### 3.2 Oral Function‐Level Staging

#### 3.2.1 Oral Function‐Level Staging (Agreement: 29/30)

*Oral function‐level staging* captures the degree of oral functional capacity based on the presence and distribution of natural teeth and/or fixed restorations (bridges, implant‐retained crowns etc.). Oral function is a critical determinant of nutritional status, oral health‐related quality of life, and the ability to maintain oral and general health in older adults. The staging is based on the concept of the shortened dental arch, which provides sufficient function for many individuals, and the presence or absence of bilateral occlusal support in the residual dentition [12]. This approach is simple, reproducible, and can be readily applied in both clinical and research settings without requiring extensive technical assessments.

**Table:** 

Stage	Criteria
Stage I	At least a shortened dental arch
Stage II	No shortened dental arch, but bilateral occlusal support on posterior teeth
Stage III	No shortened dental arch, no bilateral occlusal support on posterior teeth

Guideline for application

- *Scope of use*: Clinical function‐level staging should be applied to assess an individual's oral functional capacity, serving as an indicator of both potential prosthetic care needs and supportive care requirements.
- *Procedure*: Evaluate the dentition for the presence of a shortened dental arch and bilateral occlusal support. Staging is based on natural teeth and fixed prosthetic replacements; removable prostheses are not considered. Assign the appropriate stage (I–III) to reflect the level of clinically derived oral function.

#### 3.2.2 Subjective Function‐Level Staging (Agreement: 27/27)

*Subjective function‐level staging* incorporates the patient's own perception of how their oral conditions affect their daily functions such as eating, speaking, smiling, or comfort. This patient‐reported dimension complements clinical assessments by capturing the lived experience of oral health, which is closely linked to quality of life and care needs and priorities.

**Table:** 

Stage	Criteria
Stage I	The patient reports no oral impacts, for example, that their mouth is not adversely impacting their oral functions
Stage II	The patient reports one oral impact, for example, that their mouth is adversely impacting one oral function
Stage III	The patient reports that their mouth is adversely impacting more than one oral function, **or** the patient could not meaningfully answer the questions

Guideline for application

- *Scope of use*: Subjective function‐level staging should be applied alongside clinical function‐level staging to capture the patient's own experience and priorities
- *Procedure*: Ask the patient whether their oral condition affects specific functions (i.e., eating, smiling, speaking, comfort). Use a validated tool when available. If the patient cannot meaningfully respond (e.g., due to cognitive impairment), assign Stage III. The decision to assign the worst stage was made by consensus, while it notably introduces the risk of conflating cognitive and functional status.

### 3.3 Disease‐Level Staging

#### 3.3.1 Caries Staging (Agreement: 30/30)

*Caries staging* reflects the presence and severity of active dental caries, including both coronal and root surface caries. In older adults, root surface caries and retained root remnants are of particular relevance because they may influence function, risk of infection, and overall oral health burden. Staging distinguishes between cases without caries, those with caries but without pulp involvement or root remnants without infection, and those where caries has progressed to pulpal involvement or resulted in infection or root remnants.

**Table:** 

Stage	Criteria
Stage I	No teeth with active caries (coronal or root)
Stage II	Teeth with active caries (coronal or root) without pulpal involvement or signs of infection, also includes root remnants without infection
Stage III	Teeth with active caries (coronal or root) and pulpal involvement or signs of infection, also includes root remnants with infection

Guideline for application

- *Scope of use*: Caries staging should be applied to describe the severity and care needs of dental caries in older adults, informing decisions on preventive, restorative, or palliative care.
- *Procedure*: Assess all teeth for signs of active caries; identify root remnants, pulpal involvement, or infection. Assign the stage (I–III) according to the most severe finding present. Note that we do not presently recommend a specific instrument for assessing lesion activity (such as the Nyvad system [13] or ICDAS [14]).

#### 3.3.2 Periodontal Staging (Agreement: 26/28)

*Periodontal staging* assesses the severity of periodontal involvement in older adults based on probing pocket depth (PPD) and tooth mobility. This approach leans on the 2017 EFP/AAP Classification of Periodontal and Peri‐Implant Diseases and Conditions staging [15], but it has been simplified to focus on the specific impact of certain periodontal states in older individuals. For practicality, the assessment is limited to the *Ramfjord teeth* (teeth 16, 21, 24, 36, 41, 44), which are commonly used as index teeth in periodontal research and surveillance. If one of those teeth is missing, its immediate mesial neighbour should be examined instead. This provides a simplified yet representative evaluation of periodontal status without requiring full‐mouth charting, which may be challenging in older or care‐dependent patients.

Staging differentiates no or limited periodontal involvement (Stage I), the presence of deeper pockets without advanced mobility (Stage II), and advanced disease with both deep pockets and significant mobility (Stage III). Tooth mobility is assessed according to *Miller's classification*: *mobility II*—horizontal mobility exceeding 1 mm; *mobility III*—vertical mobility and/or depressibility in the socket. If probing is not possible and no mobility is observed, Stage II is applied.

**Table:** 

Stage	Criteria
Stage I	PPD with 4 mm or less probed mesial/distal at any of the Ramfjord teeth or their adjacent teeth (in case of the absence of the Ramfjord tooth) without mobility II/III
Stage II	PPD > 4 mm probed mesial/distal at any of the Ramfjord teeth or their adjacent teeth (in case of the absence of the Ramfjord tooth) without mobility II/III
Stage III	PPD > 4 mm and mobility II/III
Abbreviation: PPD, probing pocket depth.	

Guideline for application

- *Scope of use*: Periodontal staging should be applied to indicate the level of periodontal disease burden and to inform care needs.
- *Procedure*: Probe mesial/distal sites at the Ramfjord teeth (or their immediate substitutes if missing). Record the deepest PPD observed and assess tooth mobility according to Miller's classification. Assign the stage (I–III) based on the most severe finding. The decision to confine probing to specific teeth was made in consensus, while it should be noted that, given the individuals' age and past periodontitis experience, it is likely that not all Ramfjord teeth will be present. In that eventuality, adjacent teeth should be probed. We understand that, generally, probing only specific teeth (or indeed any partial recording protocol) risks underestimating disease burden, and we intend to validate this approach in future studies.

#### 3.3.3 Mucosa Staging (Agreement: 23/30)

*Mucosa staging* reflects the presence and severity of oral mucosal conditions, which are common in older adults and may substantially impact comfort, function, and general health. While many mucosal lesions are benign and manageable in routine care, others require prompt referral and specialist assessment. Staging distinguishes between healthy mucosa, non‐severe conditions such as candidiasis or pressure ulcers, and severe or oral potentially malignant disorders (OPMD) that necessitate further diagnostic work‐up. Stage III also explicitly includes severe cases of medication‐related osteonecrosis of the jaw (MRONJ), along with benign but clinically significant tumours such as ameloblastomas.

**Table:** 

Stage	Criteria
Stage I	Healthy oral mucosa
Stage II	Presence of a non‐severe oral mucosa lesion (e.g., Candida, pressure ulcer)
Stage III	Presence of a OPMD or malign disorder or otherwise severe mucosa lesion, requiring specialist diagnosis

Guideline for application

- *Scope of use*: Mucosa staging should be applied to identify oral mucosal health and lesions, ensuring timely recognition of conditions that may require preventive, therapeutic, or specialist care.
- *Procedure*: Perform a systematic visual inspection of mucosal surfaces including the floor of the mouth and the pharynx. Classification is based on the *clinical impression at the point of care*. Assign the stage (I–III) according to the most severe finding observed. At this point, we refrained from specifying specific lesions or providing a catalogue of visual cues. Implementation of oral mucosa staging should be evaluated, and the staging itself validated in future studies.
- *Referral*: Any lesion staged as Stage III likely requires specialist evaluation and, if indicated, histopathological confirmation (e.g., biopsy).

## 4 Discussion

Assessment and management of oral health in older adults have long been fragmented, with various initiatives proposing case definitions that failed to reach broad consensus. Our work responds to this gap by offering a comprehensive, systematic, and reproducible framework for staging and grading oral health in older adults. The present paper focuses on staging, which integrates person‐level, functional, and disease‐specific dimensions of oral health. Notably, the current step is the first of several. We have attempted to employ validated instruments (or their short form), but recognise that future studies are needed to validate the suggested approach.

An aggregation of stages into a single composite score was deliberately not undertaken at this point. The framework is intended to provide a comprehensive, multidimensional assessment of oral health and related factors in older adults. Only after exploratory and validation studies (see below) will it be possible to determine whether and how an aggregated index might be useful for clinical decision‐making, research, or policy applications.

A major strength of this project is the broad background of participants: the consensus group included academics researchers, policymakers, healthcare organisers, practitioners and stakeholders from dental and medical societies from diverse countries and professional backgrounds. The process was evidence‐based, drawing from eight reviews and additional targeted reviews, and followed rigorous consensus methodology with predefined thresholds for agreement. Nonetheless, several limitations must be acknowledged. First, it did not employ a theoretical model to design staging and grading. Instead, it used the available evidence and expert consensus. Second, certain groups were under‐represented, most notably caregivers, dental hygienists, and generally representatives from low‐ and middle‐income countries. In line, we did not include patients or the wider public at this stage, and this should be considered during further adoption to different contexts. Third, and as mentioned, the framework has not yet been formally validated and requires testing of its validity and reproducibility in clinical and research contexts. Moreover, while our consensus voting was robust, the sample size was necessarily limited, and future work should benefit from broader input. The current approach of staging and grading is highly granular and likely extensive in its application. Future activities of our group will focus on shortening the framework to facilitate its use in clinical practice, including rewording to allow usage by different health care provider groups (e.g., by nursing home staff etc.), recognising the interdisciplinary nature of geriatric care.

Dissemination strategies should target both academic and clinical audiences, ensuring that the framework can be applied consistently across diverse healthcare systems. Dissemination will be critical, both through full‐length versions for research and policy and through abbreviated, more concise versions tailored to clinical practice (see above). Translation into multiple languages and contextual adaptation will enhance reach and uptake.

Educational implementation is another relevant aspect. Integration of the framework into dental curricula and continuing professional development programmes will support a timely and necessary generational shift toward comprehensive geriatric oral care. Workshops with other professions—including medicine, nursing, nutrition, pharmacy, and social care—will strengthen interdisciplinary collaboration and promote integrated care.

Future research should map how the framework can be applied and validated in different contexts, including low‐ and middle‐income countries, where resources, infrastructure, and oral health needs may differ substantially. Generally, the acceptability and feasibility of implementing the framework in different care contexts should be evaluated. Validity should be assessed against clinical measures, quality of life, and health economics indicators in longitudinal studies. It should also be investigated whether staging and grading can validly and reliably guide preventive interventions and supportive care strategies. By aligning staging with current disease status and grading with future risk, clinicians should be able to better tailor preventive, active, and supportive therapies to individual patients, but a considerable amount of health services research will be required to confirm the utility of this systematic approach. It has the potential to improve patient outcomes, enhance resource allocation, and provide a foundation for value‐based care in gerodontology.

In conclusion, this framework offers a systematic and comprehensive approach to assessing and describing the oral health and function in older adults. It is intended to be applied in clinical care, education, research, surveillance, and health policy. The essential next steps include the validation and dissemination across contexts, including translations and cross‐cultural adaptations, simplified clinical versions, and integration into interdisciplinary education. By establishing a shared language and a coherent structure, the framework creates a solid foundation for advancing oral healthcare for older adults globally.

## Author Contributions

Each author made substantial contributions to the conception and design of the work and/or the acquisition, analysis, or interpretation of data; participated in drafting the manuscript or revising it critically for important intellectual content; approved the final version to be published; and agreed to be accountable for all aspects of the work.

## Funding

The consensus meeting was supported by the Gerodontology Association.

## Ethics Statement

The authors have nothing to report.

## Consent

All authors agreed to publish.

## Conflicts of Interest

The authors declare no conflicts of interest.

## Data Availability

The authors have nothing to report.
